# *Pinus mugo* Essential Oil Impairs STAT3 Activation through Oxidative Stress and Induces Apoptosis in Prostate Cancer Cells

**DOI:** 10.3390/molecules27154834

**Published:** 2022-07-28

**Authors:** Muhammed Ashiq Thalappil, Elena Butturini, Alessandra Carcereri de Prati, Ilaria Bettin, Lorenzo Antonini, Filippo Umberto Sapienza, Stefania Garzoli, Rino Ragno, Sofia Mariotto

**Affiliations:** 1 Department of Neurosciences, Biomedicine and Movement Sciences, Section of Biochemistry, University of Verona, 37134 Verona, Italy; muhammedashiq.thalappil@univr.it (M.A.T.); elena.butturini@univr.it (E.B.); alessandra.carcererideprati@univr.it (A.C.d.P.); ilaria.bettin@univr.it (I.B.); 2 Department of Drug Chemistry and Technology, Rome Center for Molecular Design, Sapienza University of Rome, 00185 Rome, Italy; lorenzo.antonini@uniroma1.it (L.A.); filippo.sapienza@uniroma1.it (F.U.S.); 3 Department of Drug Chemistry and Technology, Sapienza University of Rome, 00185 Rome, Italy; stefania.garzoli@uniroma1.it

**Keywords:** STAT3, oxidative stress, essential oil, apoptosis

## Abstract

Essential oils (EOs) and their components have been reported to possess anticancer properties and to increase the sensitivity of cancer cells to chemotherapy. The aim of this work was to select EOs able to downregulate STAT3 signaling using Western blot and RT-PCR analyses. The molecular mechanism of anti-STAT3 activity was evaluated through spectrophotometric and fluorometric analyses, and the biological effect of STAT3 inhibition was analyzed by flow cytometry and wound healing assay. Herein, *Pinus mugo* EO (PMEO) is identified as an inhibitor of constitutive STAT3 phosphorylation in human prostate cancer cells, DU145. The down-modulation of the STAT3 signaling cascade decreased the expression of anti-proliferative as well as anti-apoptotic genes and proteins, leading to the inhibition of cell migration and apoptotic cell death. PMEO treatment induced a rapid drop in glutathione (GSH) levels and an increase in reactive oxygen species (ROS) concentration, resulting in mild oxidative stress. Pretreatment of cells with *N*-acetyl-cysteine (NAC), a cell-permeable ROS scavenger, reverted the inhibitory action of PMEO on STAT3 phosphorylation. Moreover, combination therapy revealed that PMEO treatment displayed synergism with cisplatin in inducing the cytotoxic effect. Overall, our data highlight the importance of STAT3 signaling in PMEO cytotoxic activity, as well as the possibility of developing adjuvant therapy or sensitizing cancer cells to conventional chemotherapy.

## 1. Introduction

Signal transducer and transcription factor 3 (STAT3) is one of the seven members of the STAT family proteins that transmit signals of cytokines and growth factors from the cell surface to the nucleus [1]. The activation of the STAT3 signaling cascade is a fast and transient event that orchestrates multiple physiological cellular processes, including immune response, proliferation, differentiation and apoptosis. According to the canonical pathway, the binding of cytokines and growth factors to their receptors results in the activation of Janus tyrosine kinases (JAKs and TyK2) that phosphorylate specific tyrosine residues on the cytoplasmic tails of the receptor itself. Then SH2 domain of STAT3 binds to these docking sites, placing STAT3 within the proximity of active JAKs, which subsequently phosphorylate STAT3 at tyrosine 705 (Tyr705). Along with JAKs, STAT3 can also be activated by non-receptor tyrosine kinases, such as Src and ABL family proteins. The phosphorylated STAT3 monomers homo- or heterodimerize through SH2 domain interaction and translocate into the nucleus to regulate the transcription of target genes [2]. Several post-translational modifications have been identified for STAT3: some are believed to control phosphorylation and dimerization, while others affect nuclear import/export or DNA binding [3,4,5]. Although STAT3 activation normally leads to a physiological response, deregulation of this transduction cascade is involved in different inflammatory diseases and cancerous transformation. It has been observed that STAT3 is constitutively activated in many solid and hematological tumors, contributing to cancer initiation and progression, tumor growth, chemoresistance and metastasis [6,7,8,9,10]. Different pharmacological approaches able to counteract STAT3 hyperactivation induce the growth arrest and apoptosis of tumor cells in vitro as well as tumor regression in vivo, validating STAT3 as an anticancer target to be exploited in drug discovery [11,12,13].

Essential oils (EOs) are complex mixtures of volatile, lipophilic and aromatic low-molecular-mass secondary plant metabolites, usually obtained through steam distillation, that are usually associated with an important role in plant protection [14,15]. The bulk of EOs are composed of terpenes, but they also include non-terpene components such as phenols and alcohols. They have been recognized as sources of many bioactive products that exhibit various biological and immunological activities, including anti-inflammatory, antibacterial, antibiofilm, antifungal and antiviral effects [16,17,18,19]. Furthermore, EOs have been shown to exert cancer cell targeting activity to increase the efficacy of commonly used chemotherapy drugs and to possess pro-immune functions [20,21,22,23]. Despite a large body of research indicating that EOs and their active compounds may have anticancer properties, not many successful drug formulations have reached the clinical stage. Therefore, providing additional mechanistic proof of their anticancer efficacy might help to accelerate the development of successful drugs.

In this study, we first evaluated the anti-STAT3 activity of a panel of EOs in the human prostate cancer cell line DU145, which constitutively expresses active STAT3. Then, we investigated the molecular mechanism of the anti-STAT3 activity and consequent biological effects of *Pinus mugo* EO (PMEO), the most active EO among the selected anti-STAT3 EOs.

Overall, our data highlight the importance of STAT3 signaling in PMEO cytotoxic activity, as well as the possibility of developing adjuvant therapy or sensitizing cancer cells to conventional chemotherapy. Although we know that EOs are not substitutes for chemotherapy and radiotherapy, PMEO can be used in combination with cancer therapy to decrease the side effects of standard drugs.

## 2. Results

### 2.1. Essential Oils Dose-Dependently Inhibit STAT3 Tyrosine Phosphorylation in DU145 Cancer Cells

STAT3 is constitutively activated in highly malignant solid and hematological tumors, and its activation is critical for cell proliferation, angiogenesis, chemoresistance and metastasis. One of the critical steps leading to the activation of STAT3 is its phosphorylation of Tyr 705, dimerization and successive translocation into the nucleus [11].

As previously described, DU145 cells express Tyr705-phosphorylated STAT3 (pTyr^705^STAT3) [4]. To evaluate the effect of EOs on the constitutively active STAT3 signaling pathway, we treated DU145 cells with 25, 50 and 100 μg/mL of freshly prepared EOs for 1 h and performed Western blot analysis of whole-cell protein extracts.

Among 33 investigated EOs (Table S1), 12 were able to modulate tyrosine phosphorylation of STAT3 without affecting the total amount of STAT3 with different IC_50_ values. Arbitrarily, three levels of pTyr^705^STAT3 inhibition were considered to qualitatively cluster EO potencies: strong pTyr^705^STAT3 inhibition (IC_50_ < 50 µg/mL), medium pTyr^705^STAT3 inhibition (IC_50_ 50–100 µg/mL) and weak pTyr^705^STAT3 inhibition (IC_50_ > 100 µg/mL (Table 1)).

**Figure 1:**
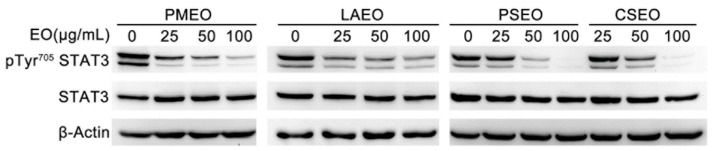
**EOs inhibit constitutive tyrosine-phosphorylated STAT3 with strong potency**. DU145 cells were treated with the indicated concentrations of EOs for 1 h, and total protein extracts were analyzed by Western blot with pTyr^705^STAT3 antibody and with anti-STAT3 antibody after membrane stripping. β-Actin is shown as the internal loading control. The data shown are representative of four independent experiments. *Pinus mugo* essential oil (PMEO), *Lavandula angustifolia* essential oil (LAEO), *Pinus sylvestris* essential oil (PSEO) and *Cupressus sempervirens* essential oil (CSEO).

Specifically, *Pinus mugo* EO (PMEO), *Lavandula angustifolia* EO (LAEO), *Pinus sylvestris* (PSEO) and *Cupressus sempervirens* (CSEO) showed strong potency (Figure 1), whereas, *Melissa officinalis* EO (MOEO), *Hyssopus officinalis* EO (HOEO), *Juniperus oxycedrus* (JOEO), *Eucalyptus globulus* (EGEO), *Chamaemelum nobile* (CNEO) and *Myrtus communis* (MCEO) showed medium potency (Figure S1a). Western blots showing the effect of 2 weak EOs are also reported in Figure S1b.

### 2.2. Essentials Oils Induce Cytotoxicity in DU145 Cells

**Figure 2:**
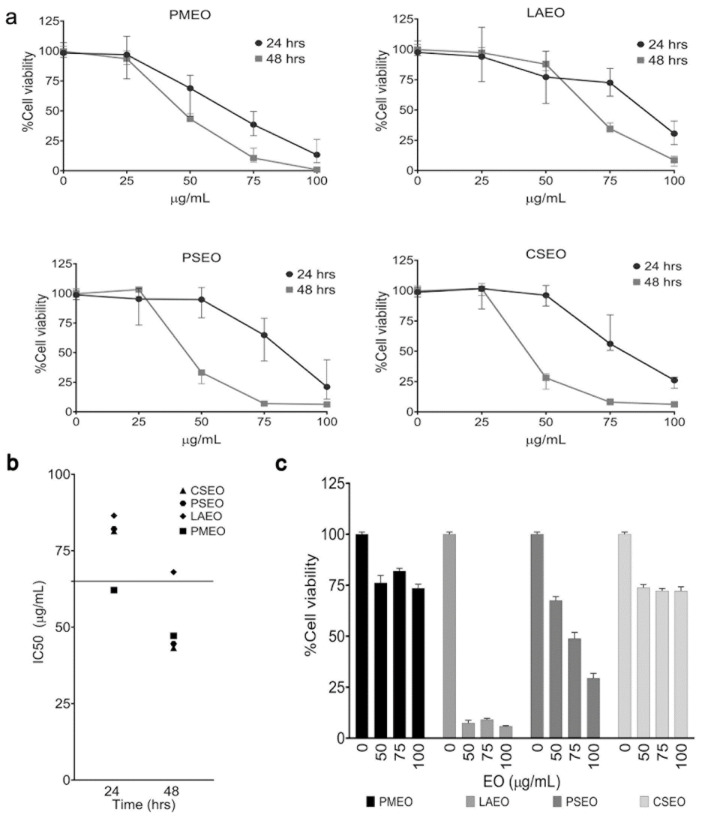
**EOs induce cytotoxicity in DU145 cell line.** DU145 cells were treated with increasing concentrations of EOs belonging to the strong cluster for 24 and 48 h, and cell viability was analyzed by WST-8 assay. (**a**) The graphs report the % viability of DU145 cells after EO treatment. The results represent the mean ± SEM value of six independent experiments. (**b**) EO doses required to affect 50% cell viability (IC50) are reported in the graph. (**c**) The graphs report the % viability of human fibroblast cells after EO treatment.

The inhibition of STAT3 activation affects cancer cell proliferation and viability. To test the cytotoxic effect of the anti-STAT3 EOs, we treated DU145 cells for 24 and 48 h with increasing concentrations of EOs belonging to the strong cluster and evaluated cell viability by WST-8 assay. As shown in Figure 2a, all four EOs in the strong cluster of anti-STAT3 activity dose-dependently affected the viability of DU145 cells in 24 h and 48 h treatments. PMEO appeared to be the most active, with IC_50_ less than 70 µg/mL at 24 h. Moreover, after 48 h treatment, three EOs, PMEO, PSEO and CSEO, exhibited high cytotoxicity, with an IC_50_ value less than 50 µg/mL. The IC_50_ values of cytotoxicity are summarized in Figure 2b.

Further, to assess whether the cytotoxicity of the EOs is specific to cancer cells, we treated non-transformed human fibroblasts with increasing concentrations of strong anti-STAT3 EOs for 24 h. As shown in Figure 2c, LAEO and PSEO strongly affected the viability of fibroblasts, whereas PMEO and MOEO showed low cytotoxicity in fibroblasts (Figure 2c).

The cytotoxic effects of EOs with medium anti-STAT3 activity on DU145 cells are shown in Figure S2.

### 2.3. Chemical Composition of Essential Oil from Pinus mugo

**Figure 3:**
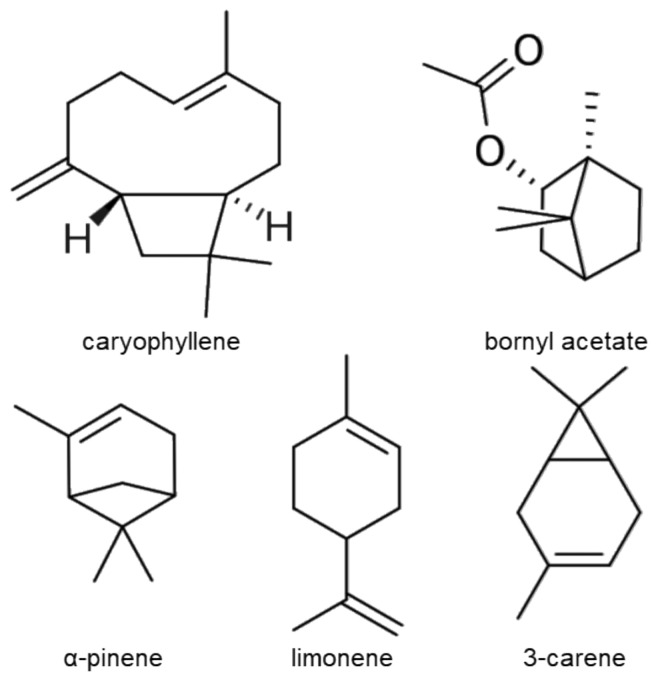
Chemical structure of main PMEO components.

The analysis of PMEO composition identified β-caryophyllene (21.4%), bornyl acetate (13.5%), α-pinene (12.5%), limonene (10.9%), δ-3-carene (10.8%), β-pinene (7.6%) and β-phellandrene (7.0%) as the most abundant chemical components (Table 2 and Figure 3).

### 2.4. Essential Oil from Pinus mugo Modulates Constitutive STAT3 Signaling

Next, we explored the anti-STAT3 activity and consequent biological effects of PMEO, which was selected as the most promising among the EOs analyzed. Indeed, this EO belongs to the strong inhibition cluster of anti-STAT3 activity, induced cytotoxicity in DU145 cells with the lowest IC_50_ value after 24 h treatment and showed minimal effects on human fibroblasts.

**Figure 4:**
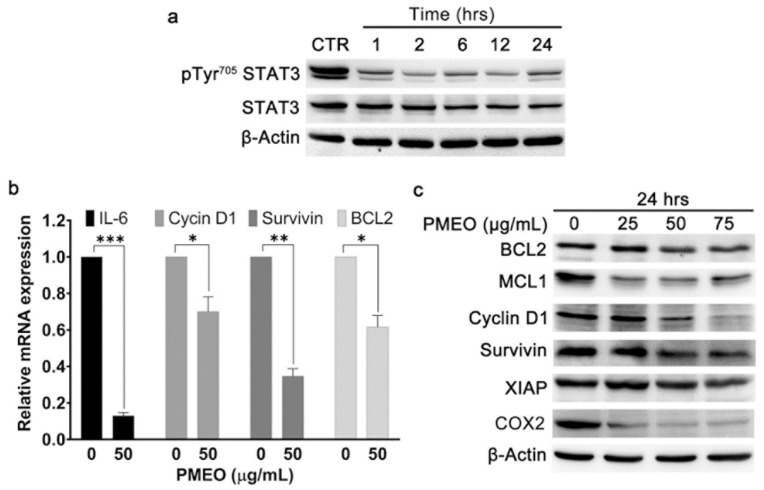
***Pinus mugo* EO modulates constitutive STAT3 signaling.** (**a**) DU145 cells were treated with 50 μg/mL PMEO for the indicated time points, and total protein extracts were analyzed by Western blot with pTyr^705^STAT3 antibody and with anti-STAT3 antibody after membrane stripping. β-Actin is shown as the internal loading control. The data shown are representative of four independent experiments. (**b**) DU145 cells were exposed to 50 μg/mL PMEO for 24 h, and total RNA was analyzed by real-time PCR assay. The data were normalized against SDHA RNA, and the levels of mRNA are expressed as the value relative to untreated cells. Each bar represents the mean ± SD of four independent experiments performed in triplicate. *p* < 0.0001 (***); *p* < 0.001 (**); *p* < 0.01(*). (**c**) DU145 cells were treated with the indicated concentrations of PMEO for 24 h, and total protein extracts were analyzed by Western blot using antibodies specific for Bcl2, MCL1, Cyclin D1, Survivin, XIAP and COX2 proteins. β-Actin is shown as the internal loading control. The data shown are representative of four independent experiments.

To analyze the kinetics of PMEO STAT3 signaling modulation, we treated DU145 cells with 50 μg/mL PMEO for 1 to 24 h. As shown in Figure 4a, PMEO treatment resulted in a time-dependent decrease in STAT3 Tyr705 phosphorylation. The maximum reduction in the levels of p^Tyr705^-STAT3 was observed within 2 h of PMEO treatment, and the signal was slightly restored after 24 h treatment.

STAT3 is a transcription factor that regulates genes involved in cell survival, proliferation, apoptosis and immune response [24]. Hence, we evaluated whether the down-modulation of STAT3 activation by PMEO also leads to the downregulation of STAT3 target genes and proteins. The incubation of DU145 cells with 50 μg/mL PMEO for 24 h significantly downregulated the mRNA expression of Cyclin D1, Bcl-2, Survivin and IL-6 (Figure 4b). Moreover, 24 h PMEO treatment resulted in decreases in the protein levels of Bcl-2, MCL1, Cyclin D1, XIAP, COX2 and Survivin (Figure 4c).

### 2.5. Essential Oil from Pinus mugo Modulates IL6-Induced STAT3 Activation

**Figure 5:**
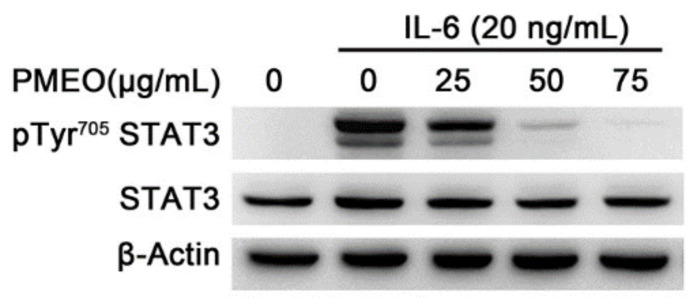
***Pinus mugo* EO modulates IL6-induced STAT3 activation**. LnCAP cells were treated with the indicated concentrations of PMEO for 45 min and then with 20 ng/mL IL-6. Total protein extracts were analyzed by Western blot using anti-pTyr^705^STAT3 antibody and anti-STAT3 antibody after membrane stripping. β-Actin is shown as the internal loading control. The data shown are representative of four independent experiments.

IL-6 plays a crucial role in maintaining normal cell growth through STAT3 activation, and its aberrant activation results in the proliferation of cancer cells. Interestingly, in the tumor microenvironment surrounding tumor cells, IL-6 is also produced by other kinds of cells, such as tumor-infiltrating immune cells and stromal cells, resulting in the hyperactivation of STAT3 signaling. STAT3 in turn promotes IL6 expression, resulting in a feedback loop that further contributes to malignancies [25]. To evaluate the effect of PMEO on IL-6-induced STAT3 signaling, the human prostate LnCAP cell line was treated with IL-6. Western blot analysis showed that 20 ng/mL IL-6 rapidly induced STAT3 Try705 phosphorylation after 15 min treatment. Pretreatment of cells for 45 min with PMEO decreased IL-6-induced Tyr705 phosphorylation of STAT3 in a dose-dependent manner, without affecting the total amount of STAT3 protein (Figure 5).

### 2.6. Essential Oil from Pinus mugo Modulates STAT3 Activation, Increasing ROS Generation

We previously identified natural compounds that enhance ROS concentration and decrease GSH level in cells. The resulting mild oxidative stress impairs STAT3 phosphorylation switching off the signaling cascade [4,5].

**Figure 6:**
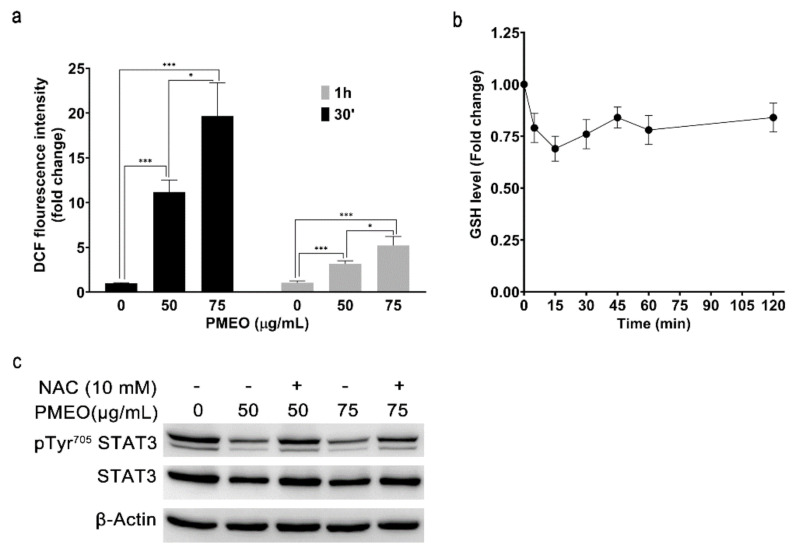
***Pinus mugo* EO induces oxidative stress and modulates STAT3 tyrosine phosphorylation.** (**a**) DU145 cells loaded with H_2_DCF-DA were treated with 50 and 75 μg/mL PMEO for 30 min or 1 h, and then the fluorescence was analyzed for ROS production. ROS levels are expressed as the value relative to untreated cells. The data are presented as means ± SEM of four independent experiments. *p* < 0.0001 (***); *p* < 0.01(*). (**b**) DU145 cells were treated with 50 µg/mL PMEO for the indicated time points, and GSH levels were spectrophotometrically analyzed by DTNB. GSH levels are expressed as the value relative to untreated cells. Data are presented as means ± SEM of five independent experiments. (**c**) DU145 cells were pretreated with 10 mM NAC for 1 h and then treated with the indicated concentrations of PMEO for 1 h more. Total protein extracts were analyzed by Western blot with pTyr^705^STAT3 antibody and with anti-STAT3 antibody after membrane stripping. β-Actin is shown as the internal loading control. The data shown are representative of four independent experiments.

To analyze whether PMEO affects the intracellular redox state, DU145 cells were loaded with the cell-permeable ROS-specific fluorescent probe H_2_DCFDA and then treated with 50 and 75 μg/mL PMEO for 30 min and 1 h. The fluorescence intensity of cells rapidly increased in a dose- and time-dependent manner, indicating the enhancement of intracellular ROS levels. We observed the maximum fold change in fluorescence intensity at 30 min compared to 1 h treatment (Figure 6a). Moreover, we evaluated the levels of intracellular GSH after PMEO treatment. Spectrophotometric analysis showed that 50 µg/mL PMEO induced a rapid and significant drop in GSH concentration (Figure 6b). Finally, to evaluate the effect of oxidative stress on the downregulation of STAT3 phosphorylation, DU145 cells were pretreated with 10 mM *N*-Acetyl-L-Cysteine (NAC), a well-known ROS scavenger, for 1 h and then treated with the indicated concentrations of PMEO for 1 h. Western blot analysis showed that NAC pretreatment partially reversed the effects of PMEO on the inhibition of STAT3 phosphorylation (Figure 6c).

### 2.7. PMEO Induces Apoptotic Death in DU145 Cells

**Figure 7:**
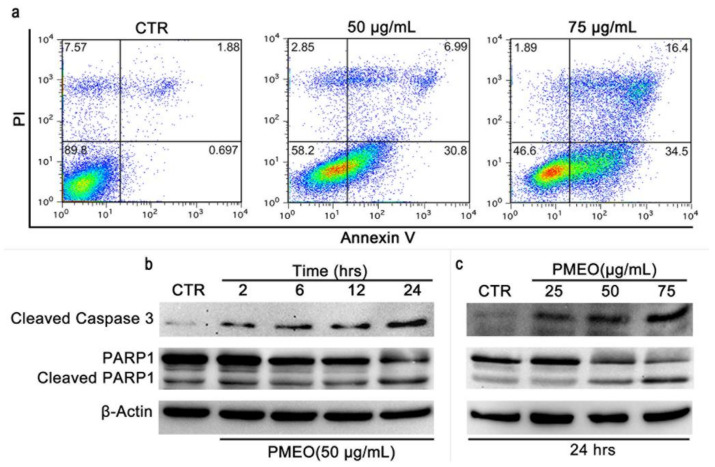
***Pinus mugo* EO induces apoptosis in DU145 cells.** (**a**) DU145 cells were treated with 50 and 75 μg/mL PMEO for 24 h, stained with Annexin V/PI and analyzed by flow cytometry for apoptosis detection. (**b**) DU145 cells were treated with 50 μg/mL PMEO for the indicated time points, and (**c**) with indicated doses for 24 h, and total protein extracts were analyzed by Western blot for the expression of cleaved caspase-3 and PARP1. PARP1 antibody recognizes both intact PARP (116 kDa) and the cleaved fragment (89 kDa). Β-Actin was used as the internal loading control. The data shown are representative of four independent experiments.

To investigate whether the loss of cell viability was related to apoptosis, PMEO-treated DU145 cells were subjected to Annexin V/PI double staining followed by flow cytometry analysis. We observed a dose-dependent increase in Annexin V^+^/PI^−^ (early apoptosis) and Annexin V^+^/PI^+^ (late apoptosis) cells, revealing the irreversible onset of the apoptotic cascade. Altogether, 38% and 50% of cells were Annexin V^+^/PI^−^ and Annexin V^+^/PI^+^, respectively, after treatment with 50 µg/mL and 75 µg/mL PMEO for 24 h (Figure 7a). The presence of caspase-3 and PARP cleavage, revealed by Western blot analysis, further confirmed the PMEO-induced apoptotic damage (Figure 7b).

### 2.8. PMEO Impairs DU145 Cell Migration

**Figure 8:**
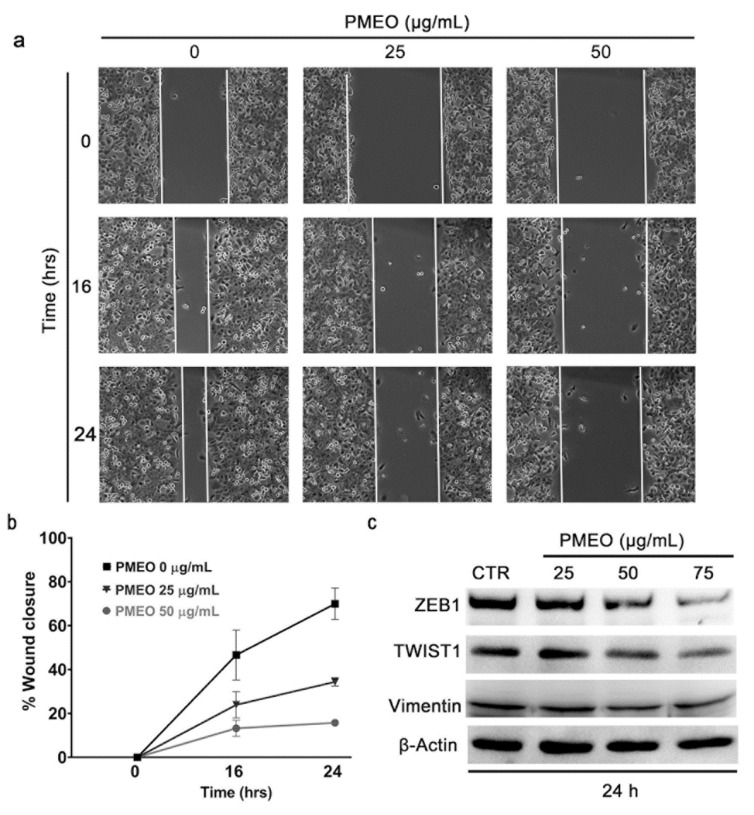
***Pinus mugo* EO inhibits cell migration in DU145 cells.** (**a**) DU145 cells were treated with 25 and 50 μg/mL PMEO for the indicated time points. (**b**) % Wound closure was calculated using ImageJ/Fiji software (https://imagej.net/Fiji). (**c**) DU145 cells were treated with 25, 50 and 75 μg/mL PMEO for 24 h, and total protein extracts were analyzed by western blot using anti-ZEB1, anti-TWIST-1 and anti-Vimentin antibodies. Β-Actin was used as the internal loading control. The data shown are representative of four independent experiments.

To investigate the effects of PMEO on cell migration, we performed a wound healing assay. Figure 8a,b demonstrate that PMEO treatment dose-dependently inhibited the migration of DU145 cells and prevented wound closure. Consistent with this result, Western blot analysis revealed that 24 h PMEO treatment dose-dependently inhibited the expression of epithelial-to-mesenchymal transition inducers ZEB1, TWIST1 and Vimentin, as well as the angiogenic factor VEGFC (Figure 8c). Altogether, these results imply the potential of PMEO to suppress cancer metastasis by impairing STAT3 signaling.

### 2.9. PMEO Acts Synergistically with Cisplatin and Enhances Chemosensitivity of DU145 Cells

**Figure 9:**
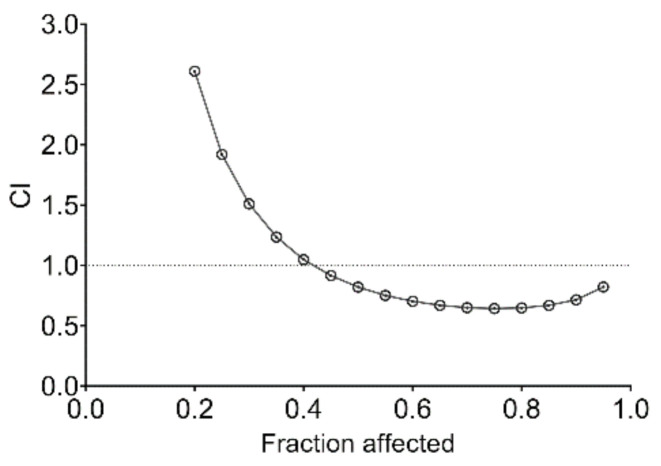
**Fa–CI plot of interaction between *Pinus mugo* EO and cisplatin.** PMEO plus cisplatin proved to be synergistic in DU145 cells.

Combination therapy has been suggested to improve the efficacy of existing chemotherapeutic drugs by overcoming chemoresistance and reducing severe side effects. After studying the efficacy of PMEO-only treatment, the cytotoxic effect of EO was evaluated in combination with cisplatin, a commonly used chemotherapeutic drug, in DU145 cells. PMEO and cisplatin were combined at a constant ratio of 2:1, and the cytotoxic effects were analyzed after 24 h treatment according to the median-effect method described by Chou and Talalay [26]. As shown in Figure 9, the combination of PMEO with cisplatin showed a slight synergistic effect.

The CIs and DRIs of drug combinations for concentrations that inhibited 50, 75, 90 and 95% of cell viability (IC_50_, IC_75_, IC_90_ and IC_95_, respectively) are reported in Table 3. The combination of PMEO and cisplatin allowed a dose reduction of cisplatin of up to 113.88 -fold (IC_95_). A favorable DRI > 1 allows a dose reduction that leads to toxicity reduction in the therapeutic applications.

## 3. Discussion

EOs are complex mixtures of hydrophobic, low-molecular-mass and volatile compounds with a broad spectrum of biological activities. Specifically, several EOs and their components are reported to exert anti-proliferative effects and induce apoptosis in cancer cells with minimal effects on non-transformed ones [27,28]. Moreover, since some of them work synergistically with commonly used anticancer drugs, they have been proposed as adjuvants in chemotherapy [20,21,22].

A lot of literature evidence describes the critical role of constitutive STAT3 signaling activation in cancer genesis and progression. Therefore, any treatment counteracting STAT3 hyperactivation has been considered a potential strategy to treat different tumors. Over the past years, various studies have also demonstrated the relation between the anti-proliferative and pro-apoptotic effects of some EOs and their anti-STAT3 activity [29,30,31,32].

In this work, we present data demonstrating the anti-STAT3 activity and consequent cytotoxic effects of a panel of EOs in human prostate cancer cells characterized by constitutive STAT3 activation. An initial screening of 33 EOs led to the selection of EOs from *Pinus mugo* (PMEO), *Lavandula angustifolia* (LAEO), *Pinus sylvestris* (PSEO) and *Cupressus sempervirens* (CSEO) as the most efficacious in the inhibition of constitutive STAT3 phosphorylation (IC_50_ < 50 µg/mL) and in the induction of cytotoxicity at 24 h (IC_50_ < 80 µg/mL). Notably, three of these EOs, PMEO, MOEO and CSEO, showed very little cytotoxicity in non-transformed human fibroblasts. This paves the way for further studies to deeply understand their mechanism of action and to validate their use as safer adjuvants to traditional chemotherapeutic agents in cancer treatment. MOEO and CSEO have previously been shown to induce apoptosis in the colon and renal adenocarcinoma cell lines, respectively [33,34,35]. Our data on the ability of these EOs to inhibit constitutive STAT3 activation are in line with these reports and suggest a possible molecular target of their cytotoxic effect. Further biochemical investigation is necessary to provide a comprehensive mechanistic understanding of the EOs described in this work.

Among the active EOs, PMEO was the most promising one, having the lowest IC_50_ in terms of anti-STAT3 activity and cytotoxicity in the DU145 cell line. We demonstrated that PMEO induces apoptosis in human prostate cancer cells without affecting the viability of non-transformed human fibroblasts. The anticancer activities of EOs from two other pine species were described earlier [36,37]. Ren et al. (2018) reported that *Pinus densiflora* needles EO suppressed tumor growth in an MCF-7 xenograft mouse model by regulating the AMPK/mTOR signaling pathway [36]. Zhang et al. (2019) reported that EO derived from *Pinus koraiensis pinecones* induced apoptosis in gastric cancer cells by regulating the HIPPO-YAP signaling pathway [37]. To the best of our knowledge, this is the first report demonstrating the anti-proliferative and pro-apoptotic effects of a pine EO mediated by the down-modulation of STAT3 signaling.

On the basis of the chemical composition, the bicyclic sesquiterpene β-caryophyllene, the oxygenated monoterpene bornyl acetate, and the monoterpenes limonene, α-pinene and δ-3-carene are the most abundant compounds of PMEO, suggesting that the anti-STAT3 activity might possibly be mediated by these compounds. β-Caryophyllene has been reported to down-modulate STAT3 signaling, thus chemosensitizing cholangiocarcinoma cells to doxorubicin [38] or sorafenib [39] treatment. Moreover, β-caryophyllene oxide has been reported to affect STAT3 signaling, thus resulting in apoptotic cell death and the inhibition of proliferation in multiple melanoma, breast and prostate cancer cell lines. However, no report exists on the anti-STAT3 activity of bornyl acetate, limonene, α-pinene and 3-carene. Nevertheless, the involvement of other minor PMEO constituents should not be ruled out. Although further studies to analyze the activity of these molecules are ongoing in our laboratory, it is important to note that EOs are considered more potent than their single components due to the synergistic and more selective effects of the mixture [40,41].

Previously, we and other researchers demonstrated that STAT3 is a redox-sensitive protein, and its activation state is related to intracellular GSH levels and ROS concentrations [2,4,42,43]. Some reports indicate that ROS trigger tyrosine phosphorylation and upregulate the DNA binding activity of STAT3 [44,45,46,47], and others point out that ROS are able to induce the oxidation of conserved cysteine residues in the DNA binding domain of STAT3, hindering its transcriptional activity [48,49]. In this regard, we identified three naturally occurring sesquiterpene lactones, dehydrocostuslactone, costunolide and cynaropicrin, able to induce mild oxidative stress, switching off the STAT3 signaling cascade in different cell lines [4,5,50]. Moreover, the consequent inhibition of the expression of proliferative and anti-apoptotic genes leads to apoptotic cell death and enhances the chemosensitivity of tumoral cells to chemotherapeutic drugs [4,5]. Herein, we report that PMEO treatment increases intracellular ROS levels and decreases intracellular GSH concentration, resulting in a prooxidant effect on cells. Decreased levels of intracellular antioxidants such as GSH, and increased ROS production are the most common event that occurs in cancer cells in response to treatment with EOs that lead to cell death. These events are temporally compatible with the data on the rapid inhibition of STAT3 phosphorylation by PMEO. Consistent with these findings, pretreatment with NAC, a well-known ROS scavenger at the time and concentration used in DU145 cells, prevents the downregulation of STAT3 phosphorylation and clarifies that oxidative stress is one of the main actors in the PMEO-induced modulation of STAT3 activation. The suppression of STAT3 activation resulted in the downregulation of its targeted gene products, which are involved in tumor survival, angiogenesis and metastasis, and thereafter led to apoptosis and impaired cellular migration. The use of natural products such as EOs that are able, on the one hand, to modulate STAT3 activation and, on the other, to induce mild oxidative stress in the highly reduced environment of cancer cells may potentially improve cancer treatment outcomes.

Chemotherapeutics such as cisplatin are effective in patients with prostate cancer, but their clinical use is limited by high toxicity and resistance development [51,52]. Therefore, new strategies are needed to develop novel therapeutic options and increase the efficacy of current treatments. Many authors suggest combination therapies using conventional chemotherapeutic agents with dietary supplements, as well as phytotherapeutic agents [53,54,55]. In this work, we showed that PMEO synergizes with cisplatin in an in vitro synergism study. The combination of PMEO with cisplatin showed better synergism within a dose range that affected more than 50% of cell viability.

In conclusion, our data provide evidence that oxidative stress induced by PMEO treatment elicits inhibitory effects on STAT3 activation and function. This induces cell death through apoptosis and chemosensitizes cells to cisplatin treatment.

## 4. Materials and Methods

### 4.1. Chemicals

All chemicals used throughout the present study were of the highest analytical grade and purchased from Merck, Milan, IT, unless otherwise specified.

### 4.2. Cell Culture

Human prostate adenocarcinoma DU145 and LnCAP cell lines (American Type Culture Collection, ATCC, Manassas, VA, USA) as well as normal primary fibroblasts (PromoCell, PBI, Milan, Italy) were maintained in Dulbecco’s modified Eagle media (DMEM, Thermo Fisher Scientific, Monza, Italy) supplemented with 10% FBS, 100 IU/mL penicillin, 100 mg/mL streptomycin and 40 mg/mL gentamycin. All cell lines were incubated at 37 °C in a humidified incubator with an atmosphere of 5% CO_2_.

### 4.3. Essential Oil Dilution

Essential oils (EOs) (Farmalabor srl, Assago, Italy) were dissolved in dimethyl-sulfoxide (DMSO) at 50 mg/mL to obtain complete solubilization and further diluted in medium for cell culture experiments, always resulting in a DMSO concentration that has no effect on cell viability.

### 4.4. GC-MS Analysis

*Pinus mugo* EO was subjected to gas chromatographic/mass spectrometric (GC/MS) analysis to characterize its composition as previously described [56]. Briefly, the GC oven program was as follows: isothermal at 60 °C for 5 min, then ramped to 220 °C at a rate of 6 °C min^−1^ and finally isothermal at 220 °C for 20 min. The identification of components was performed by matching their mass spectra with those stored in the Wiley and NIST 02 mass spectra library databases. Furthermore, the linear retention indices (LRIs) (relative to C_8_–C_30_ aliphatic hydrocarbons) were calculated and compared with available retention data presented in the literature. Relative percentages of all identified components were obtained by peak area normalization from GC-FID chromatograms without the use of an internal standard or correction factors and expressed in percentages [57]. All analyses were repeated twice.

### 4.5. Western Blot Analysis

Cells were washed with ice-cold PBS and homogenized at 4 °C in 20 mM HEPES (pH 7.4) buffer containing 420 mM NaCl, 1 mM EDTA, 1 mM EGTA, 1% Igepal, 20% glycerol, and protease and phosphatase inhibitor cocktails. Protein concentration was estimated by Coomassie Protein assay reagent (Thermo Fisher Scientific), with reference to bovine serum albumin (BSA) standards. Total protein extracts (50 µg/lane) were resolved by 7.5% or 10% SDS–polyacrylamide gel electrophoresis and transferred onto a polyvinylidene difluoride (PVDF) membrane (Immobilon P, Millipore, Bedford, MA, USA). Membranes were blocked with 5% BSA or 5% fat dry milk in Tris-buffered saline with 0.1% Tween 20 (TBST) at room temperature for 1 h and then incubated with primary antibodies specific for pTyr^705^STAT3, Cleaved Caspase-3, PARP, β-actin (Cell Signalling Technology, Beverly, MA, USA), STAT3, Cyclin D1, Survivin, TWIST1 (Santa Cruz, Santa Cruz Biotechnology, Dallas, TX, USA), XIAP, Vimentin and ZEB1 (Genetex, Irvine, CA, USA) overnight at 4 °C. After washing with TBST, the membranes were hybridized with anti-rabbit or anti-mouse IgG peroxidase-conjugated secondary antibody (Cell Signalling Technology) and developed by Western Chemiluminescent HRP Substrate (Millipore) using the ChemiDoc XRS Imaging System (Bio-Rad, Hercules, CA, USA). Blotted proteins were quantified using ImageLab 6.0.1 (BioRAd).

### 4.6. RT-qPCR Analysis

Total cellular RNA was extracted using the Pure Link RNA isolation kit (ID:12183018A, Thermo Fisher Scientific) quantified at 260/280 nm and tested by 1% agarose gel electrophoresis to check the integrity of the samples. Aliquots corresponding to 1 µg of total RNA were reverse transcribed by using the SuperScriptVilo cDNA synthesis kit (ID: 11754-(50), Thermo Fisher Scientific) following the manufacturer’s protocol. The cDNAs (corresponding to 50 ng of the original RNA) were subjected to real-time PCR with the QuantiTect SYBR Green PCR Kit (ID: 204143, Qiagen, Valencia, CA, USA) following the manufacturer’s instructions. The mRNA levels of STAT3-regulated genes, including IL-6, Bcl-2, Cyclin D1 and Survivin, were analyzed by quantitative real-time PCR. SDHA was used as the internal control.

### 4.7. Cell Viability Assay

First, 2 × 10^4^ cells were seeded in 96-well plates and left to adhere overnight. Then, cells were incubated with different concentrations of each EO, freshly prepared in DMEM, for 24 and 48 h. Cell viability was measured by the colorimetric assay based on the extracellular reduction of the tetrazolium salt 2-(2-methoxy-4-nitrophenyl)-3-(4-nitrophenyl)-5-(2,4-disulfophenyl)-2H-tetrazolium, monosodium salt (WST-8), into water-soluble formazan according to the manufacturer’s instructions (Cayman Chemical, Ann Arbor, MI, USA) [58]. The absorbance at 450 nm was measured using a microplate reader (Infinite N Nano, Tecan Trading AG, Mannendorf, Swizerland).

### 4.8. Glutathione Content Quantification

The intracellular reduced glutathione (GSH) concentration was measured by endpoint spectrophotometric titration on a Jasco V/550 spectrophotometer (JASCO, Cremella, Italy) using 5,5’-dithiobis(2-nitrobenzoic acid) (DTNB, Ellman’s reagent) [4]. Briefly, treated and untreated cells were lysed by freezing and thawing in 100 mM sodium phosphate buffer, pH 7.5, containing 5 mM EDTA (KPE buffer), and after centrifugation at 16,000 rpm for 10 min, total protein concentration was determined by using the Bradford method [59]. The supernatants were deproteinized with 5% trichloroacetic acid. For GSH measurement, acidified clear supernatants were neutralized and buffered at pH 7.4 with 200 mM K2HPO4, pH 7.5. The reaction was then started by the addition of 60 μM DTNB, and the increase in absorbance at 412 nm was measured until no variation in absorbance was evident. The amount of total GSH was determined by comparison with the GSH standard curve.

### 4.9. Measurement of Intracellular Reactive Oxygen Species

ROS production was assessed with the cell-permeable probe 2′7′-dichlorodihydrofluorescein diacetate (H_2_DCFDA; Thermo Fisher Scientific). First, 2 × 10^4^ cells were seeded in 96-well plates, left to adhere overnight and then loaded with 10 μM H_2_DCFDA. After incubation for 1 h, the cells were treated with 25 to 75 μg/mL PMEO for the indicated time. Fluorescence intensity was measured with a multimode plate reader (Ex_485 nm_ and Em_535 nm_) (Infinite N Nano, Tecan Trading). Fluorescence intensity was normalized against control wells for statistical analysis.

### 4.10. Study of Apoptotic Hallmarks

DU145 cells were treated with different concentrations of EOs for the indicated times, and three different apoptotic hallmarks were analyzed.

(a). *Dual staining with Annexin V–FITC and propidium iodide*

Cells were washed with phosphate-buffered saline and stained with Annexin V–FITC (AnxV) (Miltenyi Biotec, Bergisch Gladbach, Germany) for 15 min and propidium iodide (PI) (Thermo Fisher Scientific) immediately before acquisition on a FACS Calibur cytometer (Becton–Dickinson, Franklin Lakes, NJ, USA) [42]. PI has an elevated affinity for double-strand nucleic acids but does not enter unimpaired plasma membranes, and AnxV determines the phosphatidylserine flip from the inner to the outer leaflet of the plasma membrane. Fluorescence signals were detected on FL-1 for AnxV and on FL-3 for PI.

(b). *Caspase-3 and Poly (ADP-ribose) polymerase (PARP) cleavage*

Caspase and PARP cleavage were analyzed by Western blot as described above using an anti-PARP antibody that detects endogenous levels of full-length PARP (116 kDa) as well as the large fragment (89 kDa). After stripping, membranes were rehybridized with anti-cleaved Caspase-3 (17 kDa) and anti-actin antibodies.

### 4.11. Wound Healing Assay

DU145 cells were seeded in 6-well plates. When cells were fully confluent, a wound was created using a pipette tip to make a linear scratch through the monolayer. After washing with Hanks’ Balanced Salt Solution (HBSS), new medium containing the indicated concentration of EO was added to the designated wells. At 0, 16 and 24 h of treatment, cells were observed, and the degree of wound healing was evaluated using microscope imaging. The percentage of wound healing was analyzed using ImageJ/Fiji software version 1.53q (https://imagej.net/Fiji, accessed on 24 July 2022), applying the plugin as previously reported [60].

### 4.12. Pharmacological Synergism Studies

DU145 cells were treated with EO and cisplatin individually or in combination. The concentration of each agent that reduced cell viability by 50% (IC_50_) was preliminarily determined in the cell line to derive the constant-ratio combination design. Cytotoxicity was evaluated by WST-8 assay. The effects of interaction between EO and cisplatin were analyzed according to the median-effect method of Chou and Talalay [26] using CompuSyn software 1.0. The mean combination index (CI) values were assessed, and combination data are shown as CI vs. fraction affected (Fa) plots, defining the CI variability by the sequential deletion analysis method. CI < 1 represents synergism, CI = 1 represents an additive effect and CI > 1 represents antagonism. In addition, the dose-reduction index (DRI) for each combination was calculated. The DRI is a measure of the magnitude of dose reduction allowed for each drug when administered in synergistic combination compared with the dose of a single agent that is needed to achieve the same effect. It is considered favorable when DRI > 1.

### 4.13. Statistical Analysis

The results are expressed as the mean ± SEM of at least four independent experiments and were statistically analyzed with GraphPad Prism 8.4.2 (GraphPad Software, San Diego, CA, USA) by one-way ANOVA followed by Tukey’s multiple comparison test with the control group or within the groups. When only two groups were compared, Student’s *t*-test was used to determine significance, and *p* < 0.05 was considered statistically significant.

## Figures and Tables

**Table 1:** Essential oils classified on the basis of EC_50_ values of pTyr^705^STAT3 inhibition.

Essential Oil Plant Name	Essential Oil Acronyms	pTyr^705^STAT3 Inhibition IC50 (μg/mL)	Anti-STAT3 Potency
*Pinus mugo*	PMEO	<50	Strong
*Lavandula angustifoglia*	LAEO	<50	Strong
*Pinus sylvestris*	PSEO	<50	Strong
*Cupressus sempervirens*	CSEO	<50	Strong
*Hyssopus officinalis*	HOEO	50–100	Medium
*Juniperus oxycedrus*	JOEO	50–100	Medium
*Myrtus communis*	MCEO	50–100	Medium
*Chamaemelum Nobile*	CNEO	50–100	Medium
*Melissa officinalis*	MOEO	50–100	Medium
*Eucalyptus globulus*	EGEO	50–100	Medium
*Pimpinella anisum*	PAEO	>100	Weak
*Cananga odorata*	COEO	>100	Weak

**Table 2:** *Pinus mugus* EO chemical composition.

Components ^1^	LRI ^2^	LRI ^3^	% ^4^
α-Pinene	1018	1021	12.52
β-Pinene	1090	1099	7.63
δ-3-Carene	1142	1146	10.75
Limonene	1190	1198	10.95
β-Phellandrene	1201	1204	6.98
o-Cymene	1279	1287	2.14
α-Copaene	1492	1489	0.92
β-Cubebene	1528	1532	1.17
Linalool	1545	1547	0.18
Bornyl acetate	1466	1567	13.44
Crypton	1672	1675	3.70
β-Caryophyllene	1612	1619	21.41
Isopinocarveol	1642	1646	0.47
α-Terpineol	1650	1655	0.44
cis-Verbenol	1665	1663	0.46
Humulene	1669	1667	1.20
α-Muurolene	1733	1729	1.08
δ-Cadinene	1762	1758	1.61
Calamenene	1835	1832	0.67
p-Cymen-8-ol	1836	1838	0.85
trans-2-Caren-4-ol	1844	*	0.59
Cumaldehyde	1782	1781	0.55
SUM			99.99

^1^ The components are reported according to their elution order on a polar column; ^2^ linear retention indices measured on a polar column; ^3^ linear retention indices from the literature; * LRI not available; ^4^ percentage mean values of *Pinus mugo* EO components (%).

**Table 3:** Combination index (CI) and dose-reduction index (DRI) in synergism experiments using PMEO and cisplatin at a constant ratio.

	CI	DRI	
		Cisplatin	PMEO
IC_50_	0.82	2.59	2.31
IC_75_	0.64	10.62	1.82
IC_90_	0.72	43.59	1.44
IC_95_	0.82	113.88	1.23

## Supplementary Materials

The following supporting information can be downloaded at: https://www.mdpi.com/article/10.3390/molecules27154834/s1. Table S1: Plant name of EOs analyzed for their anti-STAT3 activity; Figure S1: Effect of EOs on constitutive tyrosine-phosphorylated STAT3; Figure S2: Effect of EOs on DU145% cell viability.

## Sample Availability

Not available.
