# Supplementary material for: Pinus mugo Essential Oil Impairs STAT3 Activation through Oxidative Stress and Induces Apoptosis in Prostate Cancer Cells
Source: Molecules. 2022 Jul 28;27(15):4834. doi: 10.3390/molecules27154834 (PMC9369512; doi:10.3390/molecules27154834)
Supplement: Supplementary file 1 [file molecules-27-04834-s001.zip › molecules-1826544-supplementary.pdf]

Pinus mugo essential oil impairs STAT3 activation through oxidative stress and induces apoptosis in prostate cancer cells

Muhammed Ashiq Thalappil[1†], Elena Butturini[1†], Alessandra Carcereri de Prati[1], Ilaria Bettin[1], Lorenzo Antonini[2], Filippo Umberto Sapieza[2], Stefania Garzoli[3], Rino Ragno[2,3,*], Sofia Mariotto[1,*]

Table S1: Plant name of EOs analyzed for their anti-STAT3 activity**.**

**Essential Oil Plant name**	**Anti-STAT3 activity**
Pinus mugo	active
Lavandula angustifoglia	active
Pinus sylvestris	active
Cupressus sempervirens	active
Hyssopus officinalis	active
Juniperus oxycedrus	active
Myrtus communis	active
Chamaemelum nobile	active
Melissa officinalis	active
Eucalyptus globulus	active
Pimpinella anisum	active
Cananga odorata	active
Salviae sclareae	Not active
Salviae officinalis	Not active
Thymus zygis	Not active
Melaleuca alternifolia	Not active
Pelargonium graveolens	Not active
Origanum vulgaris	Not active
Elettaria cardamomo	Not active
Citrus grandis	Not active
Abies sibirica	Not active
Cinnamomum camphora	Not active
Citrus aurantium amara	Not active
Citrius bergamia	Not active
Juniperus comunis	Not active
Thuja occidentalis	Not active
Citrus limon	Not active
Satureja hortensis	Not active
Citrus sinensis	Not active
Melaleuca leucadendra	Not active
Menthae piperitae	Not active
Origani majoranae	Not active
Ocimum basilicum	Not active

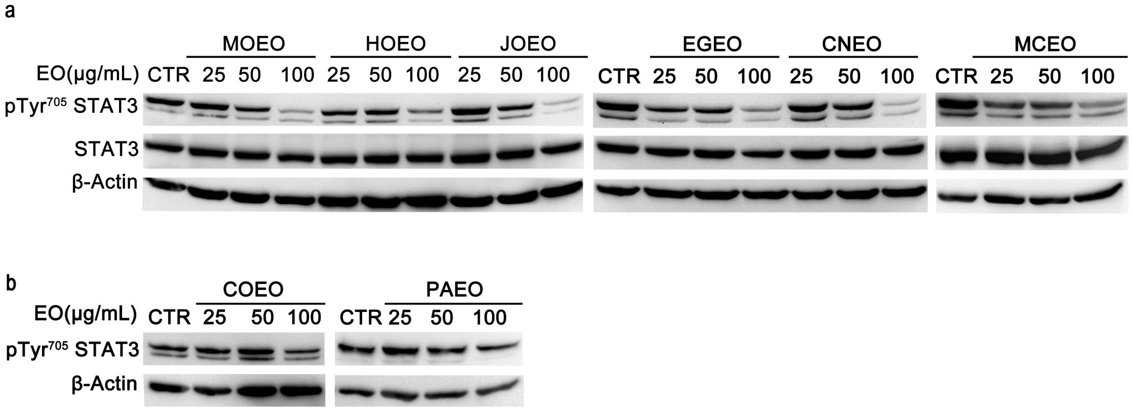

Figure S1. **Effect of EOs on constitutive tyrosine phosphorylated STAT3** DU145 cells were treated with the indicated concentration of EOs for 1 hr and total protein extracts were analyzed by western blot with pTyr[705]STAT3 antibody and, with anti-STAT3 antibody after membrane stripping. β-actin is shown as internal loading control. **(a)** EOs that inhibit STAT3 phosphorylation with medium potency. **(b)** EOs that inhibit STAT3 phosphorylation with weak potency. The data shown are representative of four independent experiments.

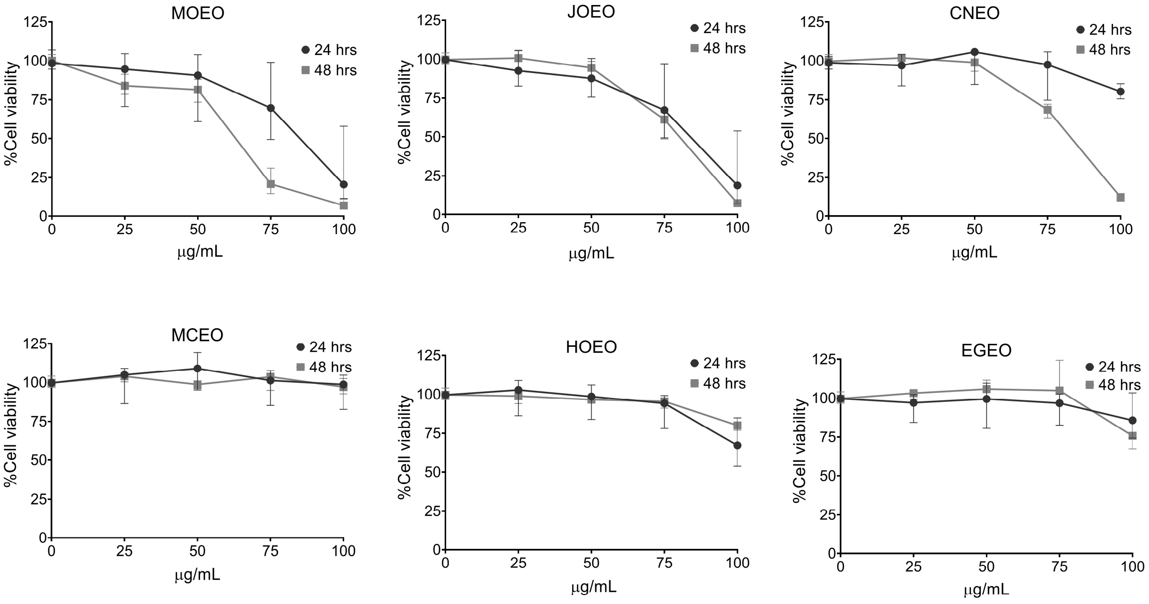

Figure S2. Effect of EOs on DU145% cells viability. DU145 cells were treated with increasing concentrations of EOs belonging the medium clusters for 24 and 48 h and the cell viability was analyzed by WST-8 assay. The graphs report the % viability of DU145 cells after EOs treatment. The results represent the mean ± SEM value of six independent experiments.
